# *N*-(Pyrrolidin-1-ylcarbothio­yl)benzamide

**DOI:** 10.1107/S1600536811053694

**Published:** 2011-12-21

**Authors:** Aisha A. Al-abbasi, Mohamed Ibrahim Mohamed Tahir, Mohammad B. Kassim

**Affiliations:** a School of Chemical Sciences & Food Technology, Faculty of Science & Technology, Universiti Kebangsaan Malaysia, 43600 Bangi, Selangor, Malaysia; b Department of Chemistry, Faculty of Science, Universiti Putra Malaysia, 43400 UPM Serdang, Selangor, Malaysia; c Fuel Cell Institute, Universiti Kebangsaan Malaysia, 43600 Selangor, Malaysia

## Abstract

In the title compound, C_12_H_14_N_2_OS, the pyrrolidine ring adopts an envelope conformation with the C atom at the 3-position as the flap and makes a dihedral angle of 65.80 (9)° with the benzene ring. In the crystal, N—H⋯O hydrogen bonds join *c*-glide related mol­ecules into chains extended along [001] that are further connected into (100) layers *via* C—H⋯O inter­actions.

## Related literature

For related compounds, their structural parameters and chemical properties, see: Al-abbasi *et al.* (2010 ▶, 2011 ▶); Al-abbasi & Kassim (2011 ▶); Ngah *et al.* (2006 ▶).
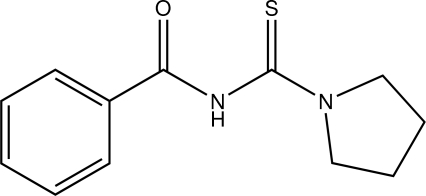


## Experimental

### 

#### Crystal data

C_12_H_14_N_2_OS
*M* *_r_* = 234.31
Monoclinic, 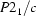

*a* = 10.3666 (4) Å
*b* = 14.6008 (5) Å
*c* = 7.8240 (3) Å
β = 98.446 (4)°
*V* = 1171.40 (8) Å^3^
*Z* = 4
Cu *K*α radiation
μ = 2.29 mm^−1^
*T* = 150 K
0.13 × 0.06 × 0.03 mm

#### Data collection

Oxford Diffraction Gemini area-detector diffractometer
Absorption correction: multi-scan (*CrysAlis RED*; Oxford Diffraction, 2006 ▶) *T*_min_ = 0.870, *T*_max_ = 0.934
8077 measured reflections
2245 independent reflections
1958 reflections with *I* > 2σ(*I*)
*R*_int_ = 0.024

#### Refinement

*R*[*F* ^2^ > 2σ(*F* ^2^)] = 0.035
*wR*(*F* ^2^) = 0.098
*S* = 1.03
2245 reflections
145 parameters
H-atom parameters constrained
Δρ_max_ = 0.32 e Å^−3^
Δρ_min_ = −0.27 e Å^−3^

### 

Data collection: *CrysAlis CCD* (Oxford Diffraction, 2006 ▶); cell refinement: *CrysAlis RED* (Oxford Diffraction, 2006 ▶); data reduction: *CrysAlis RED*; program(s) used to solve structure: *SHELXS97* (Sheldrick, 2008 ▶); program(s) used to refine structure: *SHELXL97* (Sheldrick, 2008 ▶); molecular graphics: *SHELXTL* (Sheldrick, 2008 ▶); software used to prepare material for publication: *SHELXTL*, *PLATON* (Spek, 2009 ▶) and *publCIF* (Westrip, 2010 ▶).

## Supplementary Material

Crystal structure: contains datablock(s) I, global. DOI: 10.1107/S1600536811053694/gk2440sup1.cif

Structure factors: contains datablock(s) I. DOI: 10.1107/S1600536811053694/gk2440Isup2.hkl

Supplementary material file. DOI: 10.1107/S1600536811053694/gk2440Isup3.cml

Additional supplementary materials:  crystallographic information; 3D view; checkCIF report

## Figures and Tables

**Table 1:** Hydrogen-bond geometry (Å, °)

*D*—H⋯*A*	*D*—H	H⋯*A*	*D*⋯*A*	*D*—H⋯*A*
N1—H1⋯O1^i^	0.86	2.05	2.8637 (17)	157
C11—H11*A*⋯O1^ii^	0.97	2.52	3.339 (2)	142

Symmetry codes: (i) 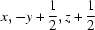; (ii) 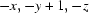.

**Fig. 1.**
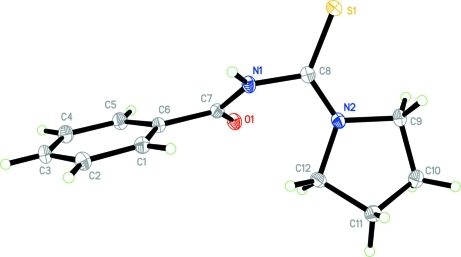
The molecular structure of the title compound with displacement ellipsods shown at the 30% probability level.

**Fig. 2.**
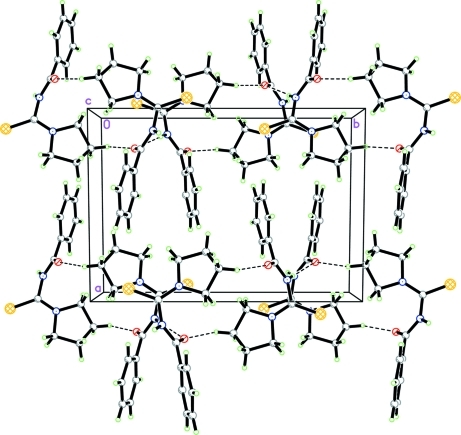
The crystal packing of the title compound viewed down the c-axis with intermolecular hydrogen bonds drawn as dashed lines.

## supplementary crystallographic information

### Comment

The title compound, I, is a derivative of a previously reported (2*S*)-1-(benzoylthiocarbamoyl)pyrrolidine-2-carboxylic acid (II) molecule (Ngah *et al.* 2006). In the crystal structure of I, the five-membered pyrrolidine ring has an envelope conformation with a maximum deviation from the mean plane of 0.238 (2)Å at C11. The benzene ring [C1/C2/C3/C4/C5/C6/C7] and the [(S1/N1/N2/C8/C9/C10] fragment are essentially planar and form dihedral angle of 79.03 (6)°.

The C=S and C=O bond lengths in I [1.6737 (17)Å and 1.2273 (19)Å, respectively] are comparable to those of II [1.662 (5)Å and 1.219 (5)Å]. In addition, the thiourea fragment in both compounds adopted a similar configuration with respect to the benzoyl and pyrrole fragments.

In the crystal, adjacent molecules are linked by N—H···O and C—H···O intermolecular interactions forming a two-dimensional polymeric structure parallel to (1 0 0) (Figure 2).

### Experimental

The title compound was synthesized according to a previously reported compound (Al-abbasi *et al.*, 2010) with some modifications. A solution of benzoyl chloride (10 mmol) in acetone was added slowly to an equimolar solution of ammonium thiocyanate in acetone. The reaction mixture was stirred at room temperature before adding piperidine (10 mmol) slowly and left stirring at room temperature for 4 h. The mixture was poured onto a water-ice and then filtered. The  product was recrystallized to give a colourless crystal, suitable for X-ray crystallography (yield 81%; m.p. 407-408 K).

### Refinement

The hydrogen atom positions were calculated geometrically and refined in a riding model approximation with C–H bond lengths in the range 0.93–0.97 Å and N–H = 0.86 Å with *U*_iso_(H) = 1.2*U*_eq_(C, N).

### Crystal data

**Table:**

C_12_H_14_N_2_OS	*F*(000) = 496
*M**_r_* = 234.31	*D*_x_ = 1.329 Mg m^−^^3^
Monoclinic, *P*2_1_/*c*	Cu *K*α radiation, λ = 1.54178 Å
Hall symbol: -P 2ybc	Cell parameters from 3639 reflections
*a* = 10.3666 (4) Å	θ = 4–71.2°
*b* = 14.6008 (5) Å	µ = 2.29 mm^−^^1^
*c* = 7.8240 (3) Å	*T* = 150 K
β = 98.446 (4)°	Needle, colourless
*V* = 1171.40 (8) Å^3^	0.13 × 0.06 × 0.03 mm
*Z* = 4	

### Data collection

**Table:**

Oxford Diffraction Gemini area-detector diffractometer	2245 independent reflections
Radiation source: fine-focus sealed tube	1958 reflections with *I* > 2σ(*I*)
graphite	*R*_int_ = 0.024
Detector resolution: 0 pixels mm^-1^	θ_max_ = 71.2°, θ_min_ = 4.3°
ω scans	*h* = −12→12
Absorption correction: multi-scan (*CrysAlis RED*; Oxford Diffraction, 2006)	*k* = −17→17
*T*_min_ = 0.870, *T*_max_ = 0.934	*l* = −9→9
8077 measured reflections	

### Refinement

**Table:**

Refinement on *F*^2^	Primary atom site location: structure-invariant direct methods
Least-squares matrix: full	Secondary atom site location: difference Fourier map
*R*[*F*^2^ > 2σ(*F*^2^)] = 0.035	Hydrogen site location: inferred from neighbouring sites
*wR*(*F*^2^) = 0.098	H-atom parameters constrained
*S* = 1.03	*w* = 1/[σ^2^(*F*_o_^2^) + (0.0577*P*)^2^ + 0.4389*P*] where *P* = (*F*_o_^2^ + 2*F*_c_^2^)/3
2245 reflections	(Δ/σ)_max_ < 0.001
145 parameters	Δρ_max_ = 0.32 e Å^−^^3^
0 restraints	Δρ_min_ = −0.27 e Å^−^^3^

### Special details

**Table:**

Experimental. The crystal was placed in the cold stream of an Oxford Cryosystems open-flow nitrogen cryostat (Cosier & Glazer, 1986) with a nominal stability of 0.1 K.(Cosier, J. & Glazer, A.*M*., 1986. *J. Appl. Cryst.*, 105, 107.)
Geometry. All e.s.d.'s (except the e.s.d. in the dihedral angle between two l.s. planes) are estimated using the full covariance matrix. The cell e.s.d.'s are taken into account individually in the estimation of e.s.d.'s in distances, angles and torsion angles; correlations between e.s.d.'s in cell parameters are only used when they are defined by crystal symmetry. An approximate (isotropic) treatment of cell e.s.d.'s is used for estimating e.s.d.'s involving l.s. planes.
Refinement. Refinement of *F*^2^ against ALL reflections. The weighted *R*-factor *wR* and goodness of fit *S* are based on *F*^2^, conventional *R*-factors *R* are based on *F*, with *F* set to zero for negative *F*^2^. The threshold expression of *F*^2^ > σ(*F*^2^) is used only for calculating *R*-factors(gt) *etc*. and is not relevant to the choice of reflections for refinement. *R*-factors based on *F*^2^ are statistically about twice as large as those based on *F*, and *R*- factors based on ALL data will be even larger.

### Fractional atomic coordinates and isotropic or equivalent isotropic displacement parameters (Å^2^)

**Table:**

	*x*	*y*	*z*	*U*_iso_*/*U*_eq_	
S1	−0.07552 (4)	0.14802 (3)	0.15309 (5)	0.02646 (15)	
O1	0.19040 (11)	0.34576 (8)	0.07829 (14)	0.0257 (3)	
N1	0.10339 (13)	0.27023 (9)	0.28912 (17)	0.0225 (3)	
H1	0.1201	0.2472	0.3911	0.027*	
N2	−0.08955 (12)	0.32949 (10)	0.14768 (16)	0.0221 (3)	
C1	0.30423 (16)	0.36102 (11)	0.5288 (2)	0.0258 (4)	
H1A	0.2228	0.3603	0.5651	0.031*	
C2	0.41496 (17)	0.38288 (13)	0.6445 (2)	0.0313 (4)	
H2	0.4074	0.3981	0.7580	0.038*	
C3	0.53634 (17)	0.38203 (13)	0.5908 (3)	0.0340 (4)	
H3	0.6104	0.3956	0.6690	0.041*	
C4	0.54804 (17)	0.36105 (13)	0.4211 (3)	0.0326 (4)	
H4	0.6300	0.3601	0.3861	0.039*	
C5	0.43831 (16)	0.34158 (12)	0.3035 (2)	0.0288 (4)	
H5	0.4459	0.3295	0.1887	0.035*	
C6	0.31615 (15)	0.34018 (11)	0.3581 (2)	0.0235 (3)	
C7	0.19885 (15)	0.31944 (11)	0.2285 (2)	0.0221 (3)	
C8	−0.02146 (15)	0.25496 (11)	0.19383 (19)	0.0215 (3)	
C9	−0.22292 (15)	0.32549 (12)	0.0516 (2)	0.0256 (3)	
H9A	−0.2807	0.2921	0.1162	0.031*	
H9B	−0.2236	0.2965	−0.0600	0.031*	
C10	−0.26313 (17)	0.42580 (13)	0.0310 (2)	0.0309 (4)	
H10A	−0.3556	0.4330	0.0351	0.037*	
H10B	−0.2434	0.4503	−0.0775	0.037*	
C11	−0.18200 (16)	0.47345 (12)	0.1844 (2)	0.0283 (4)	
H11A	−0.1726	0.5383	0.1621	0.034*	
H11B	−0.2210	0.4659	0.2889	0.034*	
C12	−0.05187 (16)	0.42453 (11)	0.1987 (2)	0.0257 (4)	
H12A	0.0028	0.4511	0.1211	0.031*	
H12B	−0.0059	0.4268	0.3159	0.031*	

### Atomic displacement parameters (Å^2^)

**Table:**

	*U*^11^	*U*^22^	*U*^33^	*U*^12^	*U*^13^	*U*^23^
S1	0.0244 (2)	0.0229 (2)	0.0312 (2)	−0.00252 (14)	0.00082 (16)	−0.00183 (14)
O1	0.0254 (6)	0.0300 (6)	0.0211 (6)	−0.0026 (4)	0.0011 (4)	0.0003 (4)
N1	0.0208 (6)	0.0272 (7)	0.0182 (6)	−0.0020 (5)	−0.0012 (5)	0.0017 (5)
N2	0.0204 (6)	0.0237 (7)	0.0214 (7)	−0.0030 (5)	0.0002 (5)	−0.0005 (5)
C1	0.0241 (8)	0.0263 (8)	0.0263 (8)	0.0006 (6)	0.0014 (7)	−0.0012 (6)
C2	0.0325 (9)	0.0311 (9)	0.0277 (9)	0.0002 (7)	−0.0039 (7)	−0.0043 (7)
C3	0.0257 (9)	0.0313 (9)	0.0403 (10)	−0.0018 (7)	−0.0105 (7)	−0.0024 (7)
C4	0.0205 (8)	0.0323 (9)	0.0447 (11)	−0.0021 (7)	0.0038 (7)	−0.0001 (8)
C5	0.0249 (8)	0.0317 (9)	0.0296 (9)	−0.0015 (6)	0.0036 (7)	−0.0018 (7)
C6	0.0220 (8)	0.0224 (8)	0.0250 (8)	−0.0005 (6)	−0.0003 (6)	−0.0002 (6)
C7	0.0207 (7)	0.0218 (8)	0.0233 (8)	0.0010 (6)	0.0016 (6)	−0.0024 (6)
C8	0.0212 (8)	0.0263 (8)	0.0173 (7)	−0.0020 (6)	0.0035 (6)	0.0003 (6)
C9	0.0193 (8)	0.0315 (9)	0.0248 (8)	−0.0027 (6)	−0.0005 (6)	−0.0012 (7)
C10	0.0250 (8)	0.0330 (10)	0.0330 (9)	0.0022 (7)	−0.0012 (7)	0.0016 (7)
C11	0.0283 (8)	0.0261 (9)	0.0299 (8)	0.0004 (6)	0.0027 (7)	0.0012 (7)
C12	0.0261 (8)	0.0219 (8)	0.0281 (8)	−0.0030 (6)	0.0001 (6)	0.0002 (6)

### Geometric parameters (Å, °)

**Table:**

S1—C8	1.6737 (16)	C4—H4	0.9300
O1—C7	1.2275 (19)	C5—C6	1.395 (2)
N1—C7	1.363 (2)	C5—H5	0.9300
N1—C8	1.413 (2)	C6—C7	1.496 (2)
N1—H1	0.8600	C9—C10	1.525 (2)
N2—C8	1.318 (2)	C9—H9A	0.9700
N2—C9	1.4746 (19)	C9—H9B	0.9700
N2—C12	1.480 (2)	C10—C11	1.528 (2)
C1—C2	1.391 (2)	C10—H10A	0.9700
C1—C6	1.392 (2)	C10—H10B	0.9700
C1—H1A	0.9300	C11—C12	1.516 (2)
C2—C3	1.384 (3)	C11—H11A	0.9700
C2—H2	0.9300	C11—H11B	0.9700
C3—C4	1.385 (3)	C12—H12A	0.9700
C3—H3	0.9300	C12—H12B	0.9700
C4—C5	1.384 (2)		
			
C7—N1—C8	123.71 (13)	N2—C8—N1	115.24 (14)
C7—N1—H1	118.1	N2—C8—S1	124.55 (12)
C8—N1—H1	118.1	N1—C8—S1	120.18 (12)
C8—N2—C9	122.08 (14)	N2—C9—C10	103.72 (13)
C8—N2—C12	126.18 (13)	N2—C9—H9A	111.0
C9—N2—C12	111.41 (13)	C10—C9—H9A	111.0
C2—C1—C6	119.56 (16)	N2—C9—H9B	111.0
C2—C1—H1A	120.2	C10—C9—H9B	111.0
C6—C1—H1A	120.2	H9A—C9—H9B	109.0
C3—C2—C1	120.06 (17)	C9—C10—C11	104.10 (13)
C3—C2—H2	120.0	C9—C10—H10A	110.9
C1—C2—H2	120.0	C11—C10—H10A	110.9
C2—C3—C4	120.29 (16)	C9—C10—H10B	110.9
C2—C3—H3	119.9	C11—C10—H10B	110.9
C4—C3—H3	119.9	H10A—C10—H10B	109.0
C5—C4—C3	120.25 (17)	C12—C11—C10	102.97 (14)
C5—C4—H4	119.9	C12—C11—H11A	111.2
C3—C4—H4	119.9	C10—C11—H11A	111.2
C4—C5—C6	119.58 (17)	C12—C11—H11B	111.2
C4—C5—H5	120.2	C10—C11—H11B	111.2
C6—C5—H5	120.2	H11A—C11—H11B	109.1
C1—C6—C5	120.22 (15)	N2—C12—C11	103.01 (13)
C1—C6—C7	121.10 (14)	N2—C12—H12A	111.2
C5—C6—C7	118.64 (15)	C11—C12—H12A	111.2
O1—C7—N1	123.00 (14)	N2—C12—H12B	111.2
O1—C7—C6	121.50 (14)	C11—C12—H12B	111.2
N1—C7—C6	115.49 (14)	H12A—C12—H12B	109.1
			
C6—C1—C2—C3	−1.3 (3)	C9—N2—C8—N1	−178.38 (13)
C1—C2—C3—C4	1.2 (3)	C12—N2—C8—N1	−5.5 (2)
C2—C3—C4—C5	0.6 (3)	C9—N2—C8—S1	−0.6 (2)
C3—C4—C5—C6	−2.1 (3)	C12—N2—C8—S1	172.25 (12)
C2—C1—C6—C5	−0.3 (3)	C7—N1—C8—N2	−59.7 (2)
C2—C1—C6—C7	−177.91 (15)	C7—N1—C8—S1	122.50 (15)
C4—C5—C6—C1	2.0 (3)	C8—N2—C9—C10	178.74 (14)
C4—C5—C6—C7	179.69 (15)	C12—N2—C9—C10	4.89 (17)
C8—N1—C7—O1	−8.9 (2)	N2—C9—C10—C11	−26.57 (16)
C8—N1—C7—C6	170.83 (14)	C9—C10—C11—C12	38.33 (17)
C1—C6—C7—O1	142.08 (16)	C8—N2—C12—C11	−154.72 (15)
C5—C6—C7—O1	−35.6 (2)	C9—N2—C12—C11	18.82 (17)
C1—C6—C7—N1	−37.7 (2)	C10—C11—C12—N2	−34.56 (16)
C5—C6—C7—N1	144.65 (16)		

### Hydrogen-bond geometry (Å, °)

**Table:**

*D*—H···*A*	*D*—H	H···*A*	*D*···*A*	*D*—H···*A*
N1—H1···O1^i^	0.86	2.05	2.8637 (17)	157
C11—H11A···O1^ii^	0.97	2.52	3.339 (2)	142
C12—H12A···O1	0.97	2.54	3.035 (2)	112

Symmetry codes: (i) *x*, −*y*+1/2, *z*+1/2; (ii) −*x*, −*y*+1, −*z*.
